# Changes in resting-state measures of prostate cancer patients exposed to androgen deprivation therapy

**DOI:** 10.1038/s41598-021-02611-6

**Published:** 2021-12-02

**Authors:** Julio Plata-Bello, Ana Plata-Bello, Yaiza Pérez-Martín, David López-Curtis, Silvia Acosta-López, Cristián Modroño, Tomás Concepción-Massip

**Affiliations:** 1 Department of Neurosurgery, Hospital Universitario de Canarias, CP 38320 S/C de Tenerife, Spain; 2 Department of Urology, Hospital Universitario de Canarias, CP 38320 S/C de Tenerife, Spain; 3 Department of Neurology, Hospital Universitario de Canarias, CP 38320 S/C de Tenerife, Spain; 4 Cognitive Neuroscience Research Group, University of La Laguna, S/C de Tenerife, Spain; 5 Department of Physiology, Faculty of Medicine, University of La Laguna, CP 38320 S/C de Tenerife, Spain; 6 Neuroscience Department, Hospital Universitario de Canarias, Calle Ofra s/n La Cuesta, La Laguna, CP 38320 S/C de Tenerife, Spain

**Keywords:** Prostate cancer, Cognitive ageing

## Abstract

The aim of the present work is to describe the differences in rs-fMRI measures (Amplitude of low frequency fluctuations [ALFF], Regional Homogeneity [ReHo] and Functional Connectivity [FC]) between patients exposed to Androgen deprivation therapy (ADT) and a control group. Forty-nine ADT patients and fifteen PC-non-ADT patients (Controls) were included in the study. A neuropsychological evaluation and a resting-state fMRI was performed to evaluate differences in ALFF and ReHo. Region of interest (ROI) analysis was also performed. ROIs were selected among those whose androgen receptor expression (at RNA-level) was the highest. FC analysis was performed using the same ROIs. Higher ALFF in frontal regions and temporal regions was identified in Controls than in ADT patients. In the ROI analysis, higher activity for Controls than ADT patients was shown in the left inferior frontal gyrus and in the left precentral gyrus. Lower ALFF in the right hippocampus and the lateral geniculate nucleus of the right thalamus was identified for Controls than ADT patients. Higher ReHo was observed in Controls in the left parietal-occipital area. Finally, ADT patients presented an increase of FC in more regions than Controls. These differences may reflect an impairment in brain functioning in ADT users.

## Introduction

Prostate cancer (PC) is the most frequent neoplasia in the male population, affecting more than 170,000 men each year in the United States alone^1^. Androgen deprivation therapy (ADT) is a common therapy for PC patients with advanced or metastatic disease^2^. The principle of ADT consists of decreasing testosterone levels by inhibiting the gonadotrophin-releasing hormone receptor (GnRHR)^3^. A reduction in testosterone has been demonstrated to improve the prognosis of PC patients with advanced or metastatic disease^4^.

ADT is associated with the development of adverse effects that may worsen the quality of life in PC patients^5^. These adverse effects can include cognitive impairment (CI) and/or dementia^6,7^. In this sense, large cohort studies and meta-analyses have found an association between the use of ADT and dementia^8–10^.

Androgens, especially testosterone and dihydrotestosterone, have been described as neuroprotective factors. Such effects have been observed in vitro using neuronal and glial cultures^11,12^. The neuroprotective effect of androgens has also been described in multiple sclerosis^13,14^ and Alzheimer’s disease^15^. Thus, the decrease in androgen levels in patients undergoing to ADT may entail reduced neuroprotection, which might be one explanation for the progressive deterioration of brain function in these patients.

Resting-state functional magnetic resonance imaging (rs-fMRI) is currently being widely used in the study of the brain. rs-fMRI is based on the presence of spontaneous low-frequency (0.01–0.08 Hz) fluctuations in the blood oxygen level–dependent (BOLD) signal^16–19^. Different approaches to the analysis of rs-fMRI data have been reported. One approach is the amplitude of low-frequency fluctuation (ALFF), an index developed by Zang et al. that enables detection of the intensity of spontaneous fluctuations, defined as the total power in the low frequency range^20^. ALFF can be considered a direct measure of functional activity in a specific brain region^21,22^, and this signal is closely related to spontaneous neural activity^23,24^. Another measure that can be extracted from rs-fMRI data is regional homogeneity (ReHo). ReHo characterizes the local synchronization of spontaneous fMRI BOLD signals and has been described as an index of local functional connectivity^25^. This method assumes that neighboring voxels within a functional cluster have similar temporal hemodynamic characteristics^26^. Finally, functional connectivity (FC) analysis is one of the most commonly used approaches to manage resting-state data. FC shows synchronization within different brain regions^16–19^ corresponding to functionally relevant resting-state networks^27–29^.

The association between all these rs-fMRI measures and cognitive-affective functions has been extensively reported^30,31^. More specifically, many studies focusing on the effect of sex steroids in rs-fMRI measures have been published (for a review, see^32^). However, only one of these addressed the changes of rs-fMRI in prostate cancer with ADT, where, as mentioned above, brain functional changes are expected after ADT onset. In this sense, the authors of this previous work described changes in the FC of the prefrontal cortex and its relationship with cognitive control measures after ADT exposure^33^.

Bearing in mind that the abovementioned previous work addressed only the changes in FC of the medial prefrontal cortex and that no studies focusing on other rs-fMRI measures have been reported in ADT recipients, we note that it would be useful to have a deeper analysis of rs-fMRI measures in ADT recipients. This may help to clarify the functional changes that are clinically evident or self-reported by the patients. Therefore, the aim of the present work is to describe the differences in rs-fMRI measures (ALFF, ReHo and FC) between a group of PC patients treated with ADT and a PC control group (not treated with ADT), both in the whole brain and in specific regions of interest with high expression of androgen receptors.

## Methods

### Patients

Forty-nine ADT patients (mean age 78.2 years [SD = 7.5]) and fifteen PC-non-ADT (control) patients (mean age 73.5 years [SD = 6.4]) were included in the study. As described in previous work^34^, all participants were right handed (using a Spanish version of the Edinburgh Handedness Inventory; http://www.neuropsicol.org/Protocol/oldfield.pdf). The patients were selected from the PC database of the Department of Urology in Hospital Universitario de Canarias (Spain). They needed to have a diagnosis of PC with a clinical indication for ADT (leuproline, triptoreline or gosereline) and they needed to have been exposed to ADT for at least 6 months. Selected ADT patients were exposed to ADT for a mean of 42.6 months (SD = 35.9). Control patients had a diagnosis of PC of more than 6 months, but without indication for ADT. Patients included in the study did not present any other psychiatric, neural or systemic disease that may have modified rs-fMRI measures and cognitive assessment. None of the patients included in the study had a history of exposure to other antiandrogen drugs. The patients included in the study had also participated in another structural study performed by the authors^34^. Among the patients included in that work, one ADT patient was excluded because of excessive head movement during rs-fMRI acquisition.

Demographic features of the ADT and Control groups are shown in Table 1. Functional status, measured with the Eastern Cooperative Oncology Group (ECOG) scale, was slightly better in ADT patients than the Controls (p = 0.042; Table 1).

**Table 1:** Clinical features of the patients included in the study.

	Control (n = 15)	ADT patients (n = 49)	p-value
Age (years)	73.5 (SD = 6.4)	78.2 (SD = 7.6)	0.163
Hypertension	60.0% (9)	69.4% (34)	0.619
Diabetes	20.0% (3)	46.9% (23)	0.205
Hypercholesterolemia	26.6% (4)	38.8% (19)	0.751
**Smoking status**			
Active smoking	6.6% (1)	8.2% (4)	0.888
History of smoking	40.0% (6)	53.1% (26)	
Metastasis	26.6% (4)	34.7% (17)	1.000
ECOG (0–1)	66.6% (10)	87.7% (43)	0.042
**Academic degree**			
No studies/primary	46.6% (7)	65.3% (32)	0.525
Secondary/superior	53.3% (8)	34.7% (17)	
Patients with at least 2 tests below − 1.5 SD	73.3% (11)	89.8% (44)	0.630
Patients with at least 1 test below − 2.0 SD	80.0% (12)	100% (49)	0.210
Depression (moderate–severe)	6.6% (1)	6.1% (3)	0.681

Continuous variables were compared using the Mann–Whitney U test, while discrete variables were compared using the Chi-Square test (level of significance p = 0.05).

Written informed consent was explained and signed by the patients and the control subjects. The study was approved by the Ethics Committee of the Hospital Universitario de Canarias, according to the Declaration of Helsinki.

### Neuropsychological assessment

A neuropsychological evaluation to determine the presence of cognitive impairment was performed by a specialist with 10 years of experience in neuropsychology. This approach was performed as it was conducted in a previous study^34^. In brief, several cognitive domains were evaluated: verbal fluency (phonetic and semantic), visuospatial and visuoperception, processing speed, visual memory and verbal memory. The tests used for the evaluation of each cognitive domain are listed in supplementary table A.1. All assessments were performed in the morning (from 8:00 am to 12:00 am). In agreement with the recommendation of the International Cognition and Cancer Task Force (ICCTF), cognitive impairment was defined when the score of at least 2 tests was equal to or below − 1.5 standard deviations (SD), or 1 test with a score equal/below − 2.0 SD. The Chi-Square test was used to compare the presence or absence of cognitive impairment (using the previous described criteria) between the studied groups. All ADT patients and 80.0% of control patients (12) presented a CI according to ICCTF criteria (Table 1)^34^.

### Data acquisition and processing

Data for the experiment were collected at the Magnetic Resonance for Biomedical Research Service of the University of La Laguna. rs-fMRI images were obtained on a 3 T General Electric (Milwaukee, WI, USA) scanner using an echo-planar imaging gradient-echo sequence and an 8 channel head coil (TR = 2000 ms, TE = 22.1 ms, flip angle = 90°, matrix size = 64 × 64 pixels, 36 slices/volume, interslice gap = 1 mm, slice thickness = 4 mm) and the same slice alignment.

A whole-brain three-dimensional structural image was acquired for anatomical reference. A 3D fast spoiled gradient—recalled pulse sequence was obtained with the following acquisition parameters: TR = 10.4 ms, TE = 4.2 ms, flip angle = 20, matrix size = 512 × 512 pixels, 0.5 × 0.5 mm in plane resolution, slice thickness = 2 mm.

After checking the images for artefacts, rs-fMRI data were preprocessed using Statistical Parametric Mapping software SPM8 (Wellcome Trust Centre for Neuroimaging; http://www.fil.ion.ucl.ac.uk/spm/). The images were spatially realigned, unwarped, and normalized to the Montreal Neurological Institute (MNI) space using standard SPM8 procedures. The first 10 images were discarded to remove signal equilibration effects. After that, the source of spurious variance was removed through linear regression by including the signal from the ventricular system, the white matter and the whole brain, in addition to the six parameters obtained by rigid body head motion correction.

All these approaches have been also used in previous works^35–37^.

### ALFF analysis

ALFF analysis was performed in a similar way as previously described^37^. The rs-fMRI Data Analysis Toolkit (REST) version 1.8 was used to perform the analysis^38^. The signal was linearly detrended and a temporal band-pass filter was applied (0.01 Hz < f < 0.08 Hz). The filtered time series was transformed to a frequency domain with a fast Fourier transform (FFT) and the power spectrum was then obtained. The square root was calculated at each frequency of the power spectrum and the averaged square root was obtained across 0.01–0.08 Hz at each voxel. Each ALFF map was spatially smoothed with a Gaussian filter of 4 mm FWHM before statistical analysis.

A two-sample t test was then performed on the ALFF maps to compare the two studied groups. Age was included as regressor of no interest to reduce the variance unrelated to the variable of interest. As the aim of the study was to study the differences between the two groups in the whole brain, a combined voxel and cluster-size thresholding approach to correct for multiple comparisons was performed. In this reagard, Monte Carlo Simulations using the AlphaSim software (included in REST), with 10,000 iterations and a voxel-wise p = 0.001, were run to determine the minimum cluster size needed to correct for Type I errors. Simulations for ALFF analysis indicated that a minimum cluster size of 16 contiguous voxels was needed to accomplish a corrected alpha of p = 0.05. (that is, 16 contiguous voxels would occur less than 5% of the time by random noise alone assuming a group of highly significantly activated voxels set at an individual voxel-wise threshold of p = 0.001).

### ReHo analysis

The ReHo maps were also generated using the REST toolkit. The signal was linearly detrended and a temporal band-pass filter was applied (0.01 Hz < f < 0.08 Hz). Kendall’s coefficient of concordance (KCC) was used to measure the similarity of the time series within a functional cluster based on the regional homogeneity hypothesis^26^. The individual ReHo maps were generated in a voxel-wise fashion, with the 27 nearest neighboring voxels defined as a cluster. The ReHo maps were divided by their own KCC value within the mask for standardization^39^. Finally, the results were included in a two-sample t test to compare the two groups. Age was included as a regressor of no interest to reduce the variance unrelated to the variable of interest. Monte Carlo Simulations (AlphaSim, 10,000 iterations, voxel-wise, p = 0.001) were run to determine the minimum cluster size needed to correct for Type I errors^39^. Simulations for ReHo analysis indicated that a minimum cluster size of 7 contiguous voxels was needed to accomplish a corrected alpha of p = 0.05.

### Region of interest (ROI) analysis

In addition to the voxel-wise analysis for ALFF and ReHo, a region of interest (ROI) analysis was also conducted in cortical and subcortical regions that had the highest expression of the androgen receptor (AR). These regions were identified using RNAseq data provided by The Allen Human Brain Atlas platform (http://human.brain-map.org/). In brief, AR expression data for five male subjects was downloaded. Only the probe of the Agilent 44 k was considered. The mean of the AR RNA expression in each considered region was calculated for the five subjects. The top-5 regions with high AR expression were selected for ROI analysis (supplementary table A.2). The selected regions were extracted from the Automated anatomical labelling atlas 3 (ALLv3)^40^. It should be noted that slight differences might exist in region-delimitation between the ALLv3 atlas and the one performed by the Allen Human Brain Atlas. Two ROIs for each region (one for each brain hemisphere) were included in the analysis. ROIs data was extracted using the MarsBaR 0.44 toolbox (http://marsbar.sourceforge.net/). The two-sample t test was performed to compare ALFF and ReHo data in each ROI between the two groups. Statistical significance was considered when the corrected p-value, using False Discovery Rate, was below 0.1 (FDR < 0.1).

### FC analysis

Functional connectivity (FC) between proximal or distant brain regions can be inferred from inter-regional cross-correlations of the BOLD signal at rest^41^. As described above, using the REST toolkit, the signal was linearly detrended and a temporal band-pass filter was applied (0.01 Hz < f < 0.08 Hz). FC analysis was performed using the previously defined ROIs. This analysis estimated the FC between each ROIs and the rest of the brain (cerebellum and grey matter, including subcortical nucleus). Individual z-score maps were obtained and the two-sample t test was then performed. The statistical significance threshold was also set using the Monte Carlo Simulations (AlphaSim, 10,000 iterations, voxel-wise, p = 0.001). Simulations for FC analysis indicated that a minimum cluster size of 6 contiguous voxels was needed to accomplish a corrected alpha of p = 0.05.

### Ethical approval

The study was approved by Hospital Universitario de Canarias ethics committee. The study was carried out in accordance with the declaration of Helsinki.

## Results

### ALFF analysis

**Figure 1:**
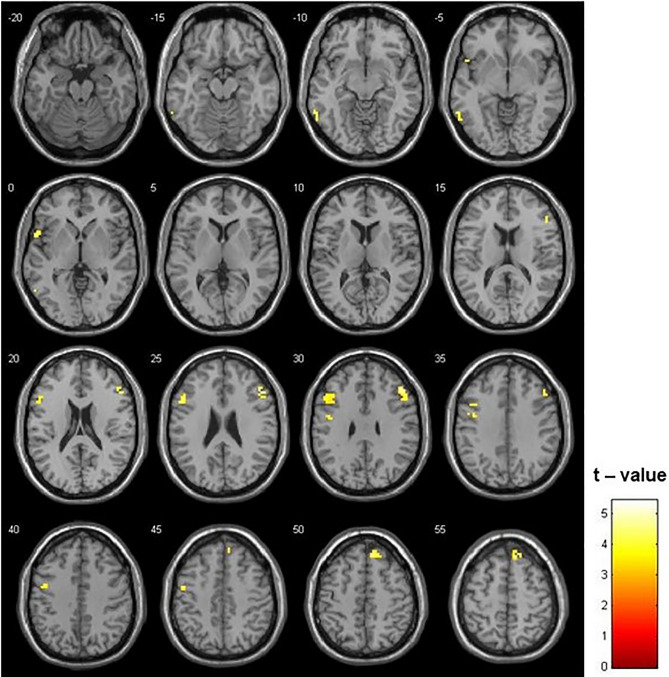
Differences between Controls and ADT patients in ALFF (p = 0.001; k = 16 voxels). Higher ALFF in frontal and temporal lobe was identified in the Controls than in ADT patients. The opposite contrast did not show any significant difference.

The two-sample t test was used to compare the ALFF maps. The control group showed a higher ALFF in frontal regions (left inferior frontal gyrus [IFG], right middle frontal gyrus and right superior frontal gyrus and left precentral gyrus [PreCG]) and temporal regions (left superior temporal gyrus and left inferior temporal gyrus) (Fig. 1, Table 2). The opposite contrast (i.e., ADT patients > Controls) did not show any significant difference with the selected threshold.

**Table 2:** Significant group differences in amplitude of low frequency fluctuation (ALFF) and regional homogeneity (ReHo).

Region	x	y	z	Cluster size	T	Z
**ALFF**						
Control patients > ADT patients						
Right middle frontal gyrus	48	30	27	57	5.41	4.85
	51	18	30		3.89	3.65
	51	33	15		3.73	3.52
Right superior frontal gyrus	12	39	51	37	4.56	4.20
	12	36	60		3.73	3.52
Left superior temporal gyrus	− 54	12	− 3	19	4.40	4.08
Left Inferior frontal gyrus	− 51	15	27	52	4.34	4.03
	− 39	12	33		3.72	3.52
Left inferior temporal gyrus	− 60	− 54	− 9	17	4.22	3.93
Left precentral gyrus	− 48	− 3	42	25	3.94	3.70
	− 45	− 3	30		3.81	3.58
ADT patients > Control patients						
–	–	–	–	–	–	–
**ReHo**						
Control patients > ADT patients						
Left superior occipital gyrus	− 42	− 81	24	27	4.84	4.42
ADT patients > Control patients						
–	–	–	–	–	–	–

Listed regions are those which survived correction for multiple comparison (p-corrected < 0.05). Coordinates are MNI coordinates.

**Figure 2:**
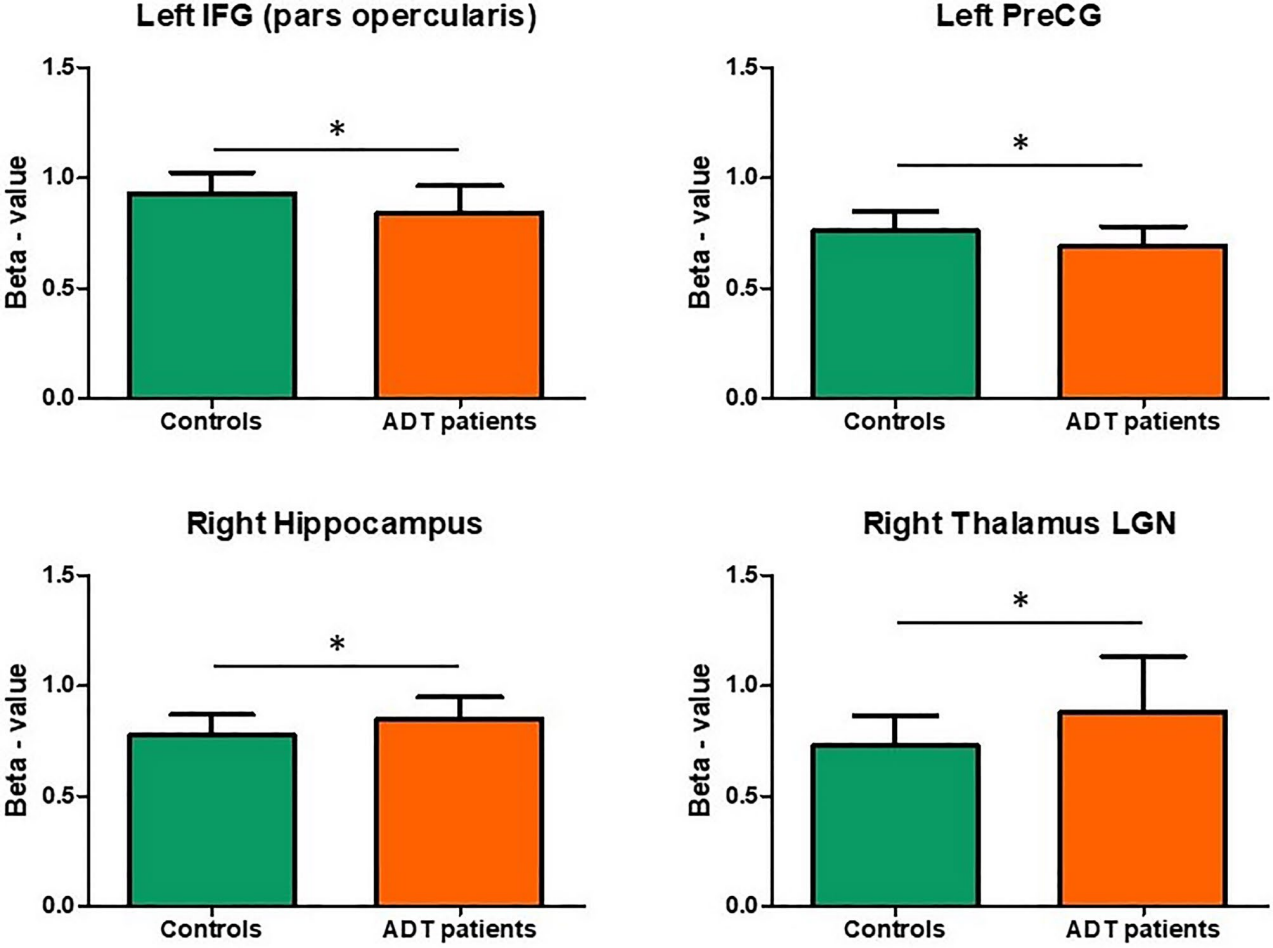
Region of interest analysis. Only the activity of regions that showed statistical significance with corrected p-values (FDR < 0.1) are plotted.

Region of Interest (ROI) analysis in ALFF maps showed a higher significant activity for Controls than ADT patients in the left IFG (pars opercularis) and in the left PreCG (Fig. 2, supplementary table A.3). The right PreCG also showed higher activity in Controls, but the corrected p-value did not reach statistical significance (FDR = 0.128). On the contrary, a higher ALFF in the right hippocampus and the lateral geniculate nucleus of the right thalamus was identified in ADT patients than Controls (Fig. 2, supplementary table A.3). The relationship between the scores of the neuropsychological tests and the ALFF in the most significant ROIs is shown in supplementary Figs. 1–7 (see supplementary material).

### ReHo analysis

**Figure 3:**
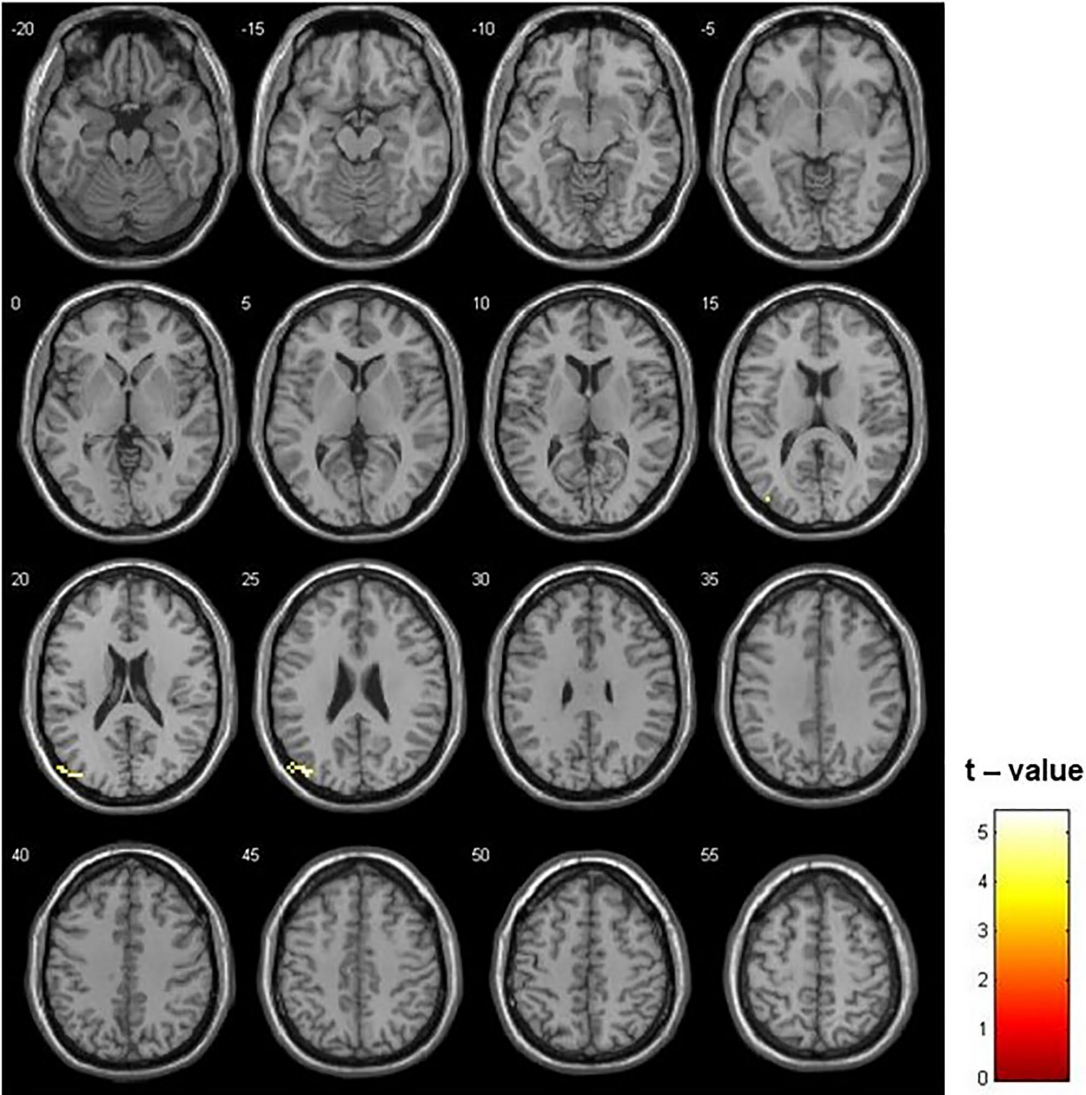
Differences between Controls and ADT in ReHo (p = 0.001; k = 7 voxels). Higher ReHo in the left superior occipital gyrus was observed in the Controls than in ADT patients. The opposite contrast did not show any significant difference.

The comparison of ReHo maps between Controls and ADT patients showed a higher ReHo in the control group in the left superior occipital gyrus (Fig. 3, Table 2). No significant differences were identified in the contrast ADT > Controls. Furthermore, no significant differences were identified between both groups of patients in the ROI analysis (supplementary table A.3).

### Functional connectivity analysis

A comparative analysis of FC in regions with high AR expression was performed between Controls and ADT patients. ADT patients showed a higher FC than Controls for many of the selected ROIs, with many other brain regions (Table 3). In other words, a higher correlation in resting-state brain activity between regions with higher AR expression and other brain areas was found in the ADT patients than in the Controls. This difference was more evident in the FC of the ventral-lateral nucleus of both thalami (largest clusters), which showed an FC with the prefrontal area (middle frontal gyrus), temporal regions (fusiform gyrus and superior temporal gyrus) and cerebellum (Table 3). Lateral geniculate nucleus of both thalami also showed higher FC with frontal areas in ADT patients, as well as both precentral and paracentral gyri which presented higher FC with close frontal-parietal areas (Table 3).

**Table 3:** Significant group differences in functional connectivity.

Region	x	y	z	Cluster Size	T	Z
**ADT patients > control patients**						
Left Amygdala						
Left Hippocampus	− 21	− 33	0	8	4.13	3.85
Right Hippocampus	18	− 33	0	14	3.69	3.48
Left IFG (Pars Opercularis)						
Left Cerebellum	− 21	− 90	− 36	9	4.31	4.00
Left Hippocampus						
Left inferior frontal gyrus (insula)	− 27	12	− 18	6	3.97	3.72
Left Paracentral lobule						
Right posterior cingulate	9	− 60	24	6	3.75	3.54
	3	− 42	27	10	3.66	3.46
Left Precentral gyrus						
Right postcentral gyrus	21	− 30	66	7	3.63	3.43
Right superior frontal gyrus	3	48	36	6	3.39	3.22
Left Thalamus (ventral lateral)						
Left middle frontal gyrus	− 36	60	− 9	29	4.77	4.37
Right middle frontal gyrus	33	63	9	27	4.70	4.31
Left fusiform gyrus	− 33	− 36	− 18	11	4.59	4.23
Right superior temporal gyrus	57	12	− 12	7	3.85	3.62
Right cerebellum	6	− 48	− 48	21	3.64	3.45
Left cerebellum	− 6	− 48	− 48		3.59	3.40
Right cerebellum	18	− 51	− 48		3.53	3.35
Right cerebellum	24	− 45	− 18	7	3.55	3.37
Left Thalamus (lateral geniculate)						
Left inferior frontal gyrus	− 12	30	− 21	9	3.67	3.47
Right Paracentral lobule						
Right cuneus	3	− 93	6	19	4.49	4.14
	3	− 87	18		4.48	4.14
Left cuneus	− 3	− 93	3	6	4.27	3.97
Left middle frontal gyrus	− 30	− 3	63	10	3.97	3.72
Right middle frontal gyrus	24	− 9	63	9	3.71	3.50
Right Precentral gyrus						
Left paracentral lobule	− 3	− 33	72	11	4.27	3.97
Right paracentral lobule	3	− 36	72	9	3.57	3.38
	6	− 48	69		3.47	3.29
Right Thalamus (ventral lateral)						
Left fusiform gyrus	− 33	− 36	− 18	6	4.40	4.08
Right superior frontal gyrus	30	66	6	15	4.11	3.83
Left middle frontal gyrus	− 36	60	− 6	16	4.02	3.76
Right Thalamus (lateral geniculate)						
Right middle frontal gyrus	30	66	9	11	4.78	4.37
Left middle frontal gyrus	− 27	36	− 15	9	3.74	3.53
Left middle frontal gyrus	− 27	63	15	7	3.63	3.44
Right Anterior Cingulate Cortex (subcallosal)						
–	–	–	–	–	–	–
**Control patients > ADT patients**						
Left Precentral gyrus						
Right inferior frontal gyrus	63	9	24	11	4.33	4.02
Right IFG (Pars Opercularis)						
Left middle temporal gyrus	− 63	− 51	6	10	4.59	4.23
Right cerebellum	54	− 84	− 33	7	3.60	3.41
Right Thalamus (lateral geniculate)						
Left insula	− 36	3	12	7	4.07	3.80
Right Anterior Cingulate Cortex (subcallosal)						
Right Insula	54	− 57	− 39	12	4.04	3.78
Right cerebellum	36	− 78	− 33	12	3.99	3.74
Left cerebellum	− 39	− 69	− 51	12	3.80	3.58
Left cerebellum	− 36	− 75	− 27	10	3.60	3.41

Listed regions are those which survived correction for multiple comparison (p-corrected < 0.05). Coordinates are MNI coordinates.

On the other hand, Controls showed higher FC than ADT patients for only a small number of regions and the main difference was located at the right subcallosal anterior cingulate cortex (ACC) (Table 3).

## Discussion

The present rs-fMRI work, using different approaches, has compared the resting-state brain activity between PC patients exposed to ADT and PC patients not exposed to ADT (Controls). On the one hand, higher ALFF and ReHo was identified in the Controls than in ADT patients in fronto-temporal and occipital areas, respectively. Furthermore, ROI analysis in regions with higher AR expression showed that some of these regions showed higher ALFF in the Controls than in ADT patients. On the other hand, the FC analysis showed a higher FC of high-AR-expression regions with other brain areas in the ADT group than in the control group. All these findings will be discussed below.

### Changes in ALFF and ReHo associated with ADT

As mentioned above, androgen exhibits a considerable influence on human behavior through the modulation of brain structure and function. Consequently, ADT may alter the brain function and this change may alter fMRI findings. In this sense, Cherrier et al. showed that ADT patients when compared to control patients (non-prostate cancer patients), after 9 months exposure to ADT, presented lower fMRI activity in the right parietal-occipital area during tasks involving manipulation and recall of spatial information^42^. Furthermore, Chao et al. described a significant decrease of brain activation during a cognitive control task in the medial prefrontal cortex, right insula and right middle/inferior frontal gyri after 6 months with ADT^33^. These results agree with another work that, using positron emission tomography (PET) techniques, demonstrated an increase in cerebral glucose metabolism during a mental rotation task in occipital and frontal regions after 12 weeks of testosterone treatment in hypogonadal patients^43^. In other words, the brain of males with low androgen levels shows a lower activation during cognitive tasks. Nonetheless, the present work is focused on resting state measures (i.e., without performing any task). Bearing this in mind, another longitudinal PET study described a positive correlation between free testosterone levels and regional cerebral blood flow (rCBF) in the hippocampus and frontal regions (right IFG and anterior cingulate gyrus)^44^ which may be associated with rs-fMRI changes found in the present work. More interestingly, a previous work, focusing on transgender people and using the same measures as the present study (i.e., ALFF and ReHo measures), demonstrated a shift of ALFF and ReHo related to androgen levels in the frontal cortex (mainly the IFG, PreCG and premotor cortex), medial temporal cortex (mainly in the parahippocampal gyrus) and in the cerebellum^39^ which are compatible with the differences between controls and ADT patients described in the present work.

In the present work, patients exposed to ADT presented a decrease ALFF during rs-fMRI in various frontal areas. More specifically, the left primary motor cortex, the premotor cortex bilaterally and the IFG bilaterally showed a higher ALFF in Controls than in ADT patients. This higher activation of frontal regions seems to be compatible with the results reported in previous studies^33,39,42–44^. Furthermore, the ROI analysis showed that, apart from the presence of ALFF differences in frontal areas, the right hippocampus and the right thalamus also showed significant differences. However, in these regions, ALFF was higher for ADT patients than Controls. This finding may indicate that the effect of testosterone in BOLD signal varies between different brain regions. In this regard, testosterone levels and AR activation in frontal areas (neocortex) would lead to an ALFF increase, while in the hippocampus (allocortex) or subcortical nuclei (thalamus) would lead to an ALFF decrease.

Considering the findings in the ReHo analysis, where higher homogeneity in parietal-occipital areas for control patients was shown, ADT seems to alter the functional status of the occipital cortex. This result is also supported by previous works. Indeed, the reduced parieto-occipital activation during manipulation and recall of spatial information tasks in ADT patients reported by^42^ showed that testosterone levels influence occipital cortex functioning. Furthermore, further evidence supporting this finding is the positive relationship between testosterone levels and cerebral glucose metabolism during a mental rotation task in occipital areas^44^.

In summary, although the present study is the first study focusing on ADT patients describing differences in ALFF and ReHo measures, the findings here are compatible with those reported in previous neuroimaging works focused on the effect of androgens on brain function. Other cancer patients (e.g. breast or lung cancer) showed also changes in ALFF and/or ReHo related to the use of chemotherapeutic agents^45,46^. Nevertheless, the finding presented here cannot be associated with chemotherapy, because it was not used in any of the selected patient before their inclusion in the study.

### ADT patients showed more diffuse FC than controls

FC reflects the relationship between different brain regions and measures how well individual brain regions activate in a concerted manner as an important index of the integrity of brain functions. Androgens have previously been shown to modulate brain connectivity. In this respect, higher testosterone levels in boys were associated with reduced FC between the amygdala and the orbito-frontal cortex^47^. This finding has also been reported elsewhere in adults^48^. The administration of a single dose of testosterone leads to a reduction in frontal-subcortical and frontal-parietal functional connectivity that potentially contributes to impaired emotion processing and regulation^49^. More evidence about the reduction of FC related to testosterone comes from anabolic steroid users. Reduced FC between the superior frontal gyrus and the dorsal attention network as well as between the amygdala and the default mode network has been described in anabolic steroid users^50^. However, the only study that performed an FC analysis in ADT patients, described a decrease in the FC of the medial prefrontal cortex in ADT patients^33^.

The main finding in the FC analysis in the present study was the presence of higher FC in ADT patients than in Controls in most of the selected ROIs. As described in the Methods section, the selected ROIs were those brain regions that have shown the highest AR-RNA expression, thus they are putatively the regions that may be more sensitive to the lack of circulating androgens. The thalamus (ventral-lateral and lateral-geniculate nuclei) and primary motor regions (PreCG and paracentral lobule) were the areas that showed more FC for ADT patients. On the contrary, the subcallosal region of the anterior cingulate cortex showed a significantly higher FC in Controls than ADT patients. As shown in the ALFF analysis, the effect of androgens in FC seems to depend on the ROI considered. Generally speaking, the present results agree with previous publications that described a general attenuation of FC with higher levels of testosterone^47–50^. The development of the anterior cingulate cortex, involved in emotion and motivation and belonging to the limbic system, is known to be influenced by sex hormones^51^. In regions that belong to the limbic system, androgens seem to induce the increase of FC with other brain regions and this might explain the anxiolytic and antidepressant effects that have been reported for testosterone^52^.

### Limitations

The main limitation of the present study is its case–control study nature. Therefore, a longitudinal study would be preferable to confirm that the described functional changes are secondary to ADT use and the temporal relationship between these changes and the onset of cognitive decline (measured by standard cognitive assessment). In this regard, if the rs-fMRI changes come early than cognitive decline measured by standard tests, the rs-fMRI information would help the clinician to make changes in the patient`s management of the (e.g. cognitive rehabilitation or changes in the ADT posology).

Furthermore, larger cohorts of ADT and non-ADT patients are also recommended to obtain stronger evidence and the effect of other drugs that are currently used in castration-resistant PC (e.g. Enzalutamide or Abiraterone) which target the AR or the testosterone synthesis, respectively, should also be analyzed in prospective studies.

Another limitation of the present work is that the ADT group showed an older age distribution and the effect of the variability in preprocessing and statistical analysis performed here compared to other neuroimaging studies cannot be estimated.

Finally, the clinically-significant ALFF or ReHo changes cutoff and the reversibility of the rs-fMRI changes once the ADT has been stopped should also be studied in longitudinal-prospective studies.

## Conclusion

Differences in rs-fMRI measures have been identified between ADT patients and Controls. Firstly, higher ALFF was identified in Controls than in ADT patients in frontal–temporal regions, although ALFF seems to increase in the right hippocampus and right thalamus in ADT patients. ReHo analysis showed a higher homogeneity in parietal-occipital areas in Controls than in ADT patients. Finally, FC was generally higher in ADT patients compared to Controls.

## Supplementary Information

Supplementary Information.

## Data Availability

The datasets generated during the current study are available from the corresponding author on reasonable request.
