# Supplementary material for: Changes in resting-state measures of prostate cancer patients exposed to androgen deprivation therapy
Source: Sci Rep. 2021 Dec 2;11:23350. doi: 10.1038/s41598-021-02611-6 (PMC8639725; doi:10.1038/s41598-021-02611-6)

**Title**

Changes in Resting-State Measures of Prostate Cancer Patients Exposed to Androgen Deprivation Therapy

**Authors**

Julio Plata-Bello*^1,4^, Ana Plata-Bello^2,4^, Yaiza Pérez-Martín^3^, David López-Curtis^4^, Silvia Acosta-López^4^, Cristián Modroño^5^, Tomás Concepción-Massip^2^.

**Affiliation**

1 Hospital Universitario de Canarias (Department of Neurosurgery), S/C de Tenerife, Spain. CP 38320.

2 Hospital Universitario de Canarias (Department of Urology), S/C de Tenerife, Spain. CP 38320.

3 Hospital Universitario de Canarias (Department of Neurology), S/C de Tenerife, Spain. CP 38320.

4 Cognitive Neuroscience Research Group from University of La Laguna, Spain.

5 Department of Physiology, Faculty of Medicine, University of La Laguna, Spain. CP 38320.

**Corresponding author**

Julio Plata-Bello

Address: Hospital Universitario de Canarias (Neuroscience department), Calle Ofra s/n La Cuesta. CP 38320. La Laguna, S/C de Tenerife. Spain. Phone number: +34 922 255 544 / +34 646 625 973. E – mail address: jplabel@gobiernodecanarias.org

| **Table A.1.** List of cognitive tests used for the cognitive assessment. | |
| --- | --- |
| **Cognitive domain** | **Test(s)** |
| **Verbal fluency** | *Word List Generation (WLG)*  *Controlled Oral Word Association Test (COWAT)* |
| **Visuospatial and visuoperceptive** | *Hooper Organization Visual Test (HOVT)*  *Judgement Line Orientation Test (JLOT)* |
| **Processing speed** | *Trail Making Test Part A (TMT A)* |
| **Visual memory** | *Brief Visuospatial Memory Test (BVMT)* |
| **Verbal memory** | *Auditive Verbal Spanish Complutense Test (TAVEC)* |
| **Dependency** | *Lawton & Brody scale* |
| **Mood assessment** | *Beck Depression Inventory-II (BDI-II)* |

| **Table A.2. Cortical and subcortical regions with highest androgen receptor expression (at RNA level) and their corresponding label in the Automated anatomical labelling atlas 3 (AAL v3).** | | | |
| --- | --- | --- | --- |
| **Region from The Allen Human Brain Atlas** | **AR-RNA mean expression (log2[1+x])** | **SD** | **ROI using the AAL v3** |
| Inferior frontal gyrus, opercular part | 0.1431 | 0.1673 | Frontal_Inf_Oper_L  Frontal_Inf_Oper_R |
| Precentral gyrus | 0.4128 | 0.2441 | Precentral_L  Precentral_R |
| Subcallosal Cingulate gyrus | 0.314 | 0.3939 | ACC_sub_L  ACC_sub_R |
| CA2 field | 1.9556 | 0.3683 | Hippocampus_l  Hippocampus_R |
| CA3 field | 1.6133 | 0.4277 |  |
| CA4 field | 1.4957 | 0.3434 |  |
| Piriform cortex | 0.2779 | 0.8978 | Amygdala_L  Amygdala_R |
| Paracentral lobule, anterior part | 0.1728 | 0.1510 | Paracentral_Lobule_L  Paracentral_Lobule_R |
| Dorsal lateral Geniculate nucleus | 1.7979 | 0.6126 | Thal_LGN_L  Thal_LGN_R |
| Lateral group of nuclei, ventral division | 1.7231 | 0.4645 | Thal_VL_L  Thal_VL_R |

| **Supplementary table A.3. Region of interest analysis in selected regions where the androgen receptor is highly expressed. Means and standard deviations (SD) are related to beta-values for each region. Statistical significance is considered when FDR<0.1.** | | | | | | | |
| --- | --- | --- | --- | --- | --- | --- | --- |
| **ALFF** | | | | | | | |
| **Region** | **Control** | | **ADT** | | **T** | **p-value** | **FDR** |
|  | **Mean** | **SD** | **Mean** | **SD** |  |  |  |
| *Frontal_Inf_Oper (left)* | 0.93 | 0.09 | 0.84 | 0.12 | 2.38 | 0.020 | **0.080** |
| *Frontal_Inf_Oper (right)* | 0.89 | 0.13 | 0.81 | 0.12 | 1.92 | 0.059 | 0.157 |
| *Precentral (left)* | 0.76 | 0.09 | 0.69 | 0.09 | 2.50 | 0.015 | **0.080** |
| *Precentral (right)* | 0.70 | 0.11 | 0.64 | 0.09 | 2.10 | 0.040 | 0.128 |
| *Hippocampus (left)* | 0.83 | 0.10 | 0.87 | 0.11 | -1.31 | 0.196 | 0.348 |
| *Hippocampus (right)* | 0.77 | 0.09 | 0.85 | 0.09 | -2.60 | 0.012 | **0.080** |
| *Amygdala (left)* | 0.95 | 0.33 | 1.02 | 0.37 | -0.62 | 0.537 | 0.661 |
| *Amygdala (right)* | 0.97 | 0.27 | 1.03 | 0.35 | -0.62 | 0.534 | 0.661 |
| *Paracentral (left)* | 0.84 | 0.16 | 0.85 | 0.19 | -0.17 | 0.862 | 0.862 |
| *Paracentral (right)* | 0.80 | 0.17 | 0.76 | 0.16 | 0.82 | 0.416 | 0.661 |
| *Thal_VL (left)* | 0.84 | 0.12 | 0.87 | 0.18 | -0.67 | 0.504 | 0.661 |
| *Thal_VL (right)* | 0.82 | 0.15 | 0.85 | 0.17 | -0.52 | 0.602 | 0.688 |
| *Thal_LGN (left)* | 0.77 | 0.10 | 0.89 | 0.25 | -1.71 | 0.092 | 0.184 |
| *Thal_LGN (right)* | 0.73 | 0.13 | 0.88 | 0.25 | -2.93 | 0.006 | **0.080** |
| *ACC_sub (left)* | 1.03 | 0.17 | 1.16 | 0.37 | -1.80 | 0.079 | 0.181 |
| *ACC_sub (right)* | 0.94 | 0.19 | 0.96 | 0.26 | -0.29 | 0.771 | 0.822 |
| **ReHo** | | | | | | | |
| **Region** | **Control** | | **ADT** | | **T** | **p-value** | **FDR** |
|  | **Mean** | **SD** | **Mean** | **SD** |  |  |  |
| *Frontal_Inf_Oper (left)* | 0.92 | 0.07 | 0.91 | 0.07 | 0.80 | 0.436 | 0.797 |
| *Frontal_Inf_Oper (right)* | 0.90 | 0.06 | 0.89 | 0.09 | 0.39 | 0.703 | 0.803 |
| *Precentral (left)* | 0.94 | 0.04 | 0.91 | 0.07 | 1.63 | 0.114 | 0.656 |
| *Precentral (right)* | 0.94 | 0.06 | 0.93 | 0.07 | 0.55 | 0.590 | 0.797 |
| *Hippocampus (left)* | 1.00 | 0.08 | 1.02 | 0.10 | -0.67 | 0.510 | 0.797 |
| *Hippocampus (right)* | 0.99 | 0.08 | 1.00 | 0.09 | -0.41 | 0.688 | 0.803 |
| *Amygdala (left)* | 0.89 | 0.19 | 0.88 | 0.15 | 0.09 | 0.928 | 0.972 |
| *Amygdala (right)* | 0.87 | 0.14 | 0.88 | 0.15 | -0.04 | 0.972 | 0.972 |
| *Paracentral (left)* | 0.96 | 0.09 | 0.99 | 0.09 | -1.05 | 0.306 | 0.797 |
| *Paracentral (right)* | 0.98 | 0.09 | 1.00 | 0.09 | -0.82 | 0.422 | 0.797 |
| *Thal_VL (left)* | 1.15 | 0.15 | 1.22 | 0.11 | -1.89 | 0.063 | 0.656 |
| *Thal_VL (right)* | 1.15 | 0.17 | 1.20 | 0.11 | -1.41 | 0.164 | 0.656 |
| *Thal_LGN (left)* | 1.09 | 0.18 | 1.15 | 0.14 | -1.49 | 0.141 | 0.656 |
| *Thal_LGN (right)* | 1.11 | 0.17 | 1.13 | 0.17 | -0.53 | 0.598 | 0.797 |
| *ACC_sub (left)* | 0.95 | 0.14 | 0.98 | 0.14 | -0.68 | 0.500 | 0.797 |
| *ACC_sub (right)* | 0.99 | 0.16 | 1.02 | 0.16 | -0.60 | 0.549 | 0.797 |

**Supplementary figure 1. Interaction analysis between ALFF in selected ROIs and Word List Generation (WLG) score.**


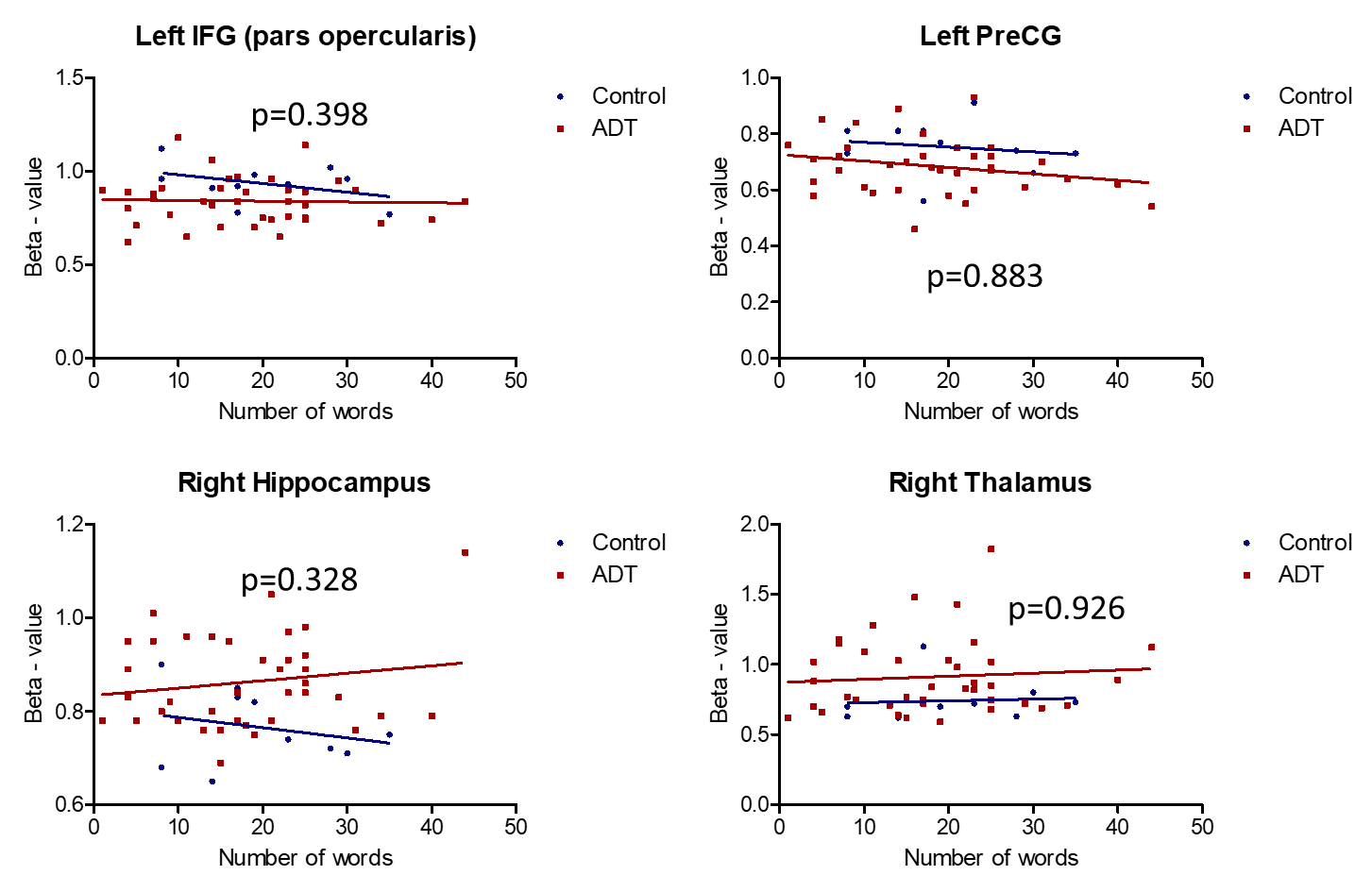


**Supplementary figure 2.** **Interaction analysis between ALFF in selected ROIs and Controlled Oral Word Association Test (COWAT) score.**


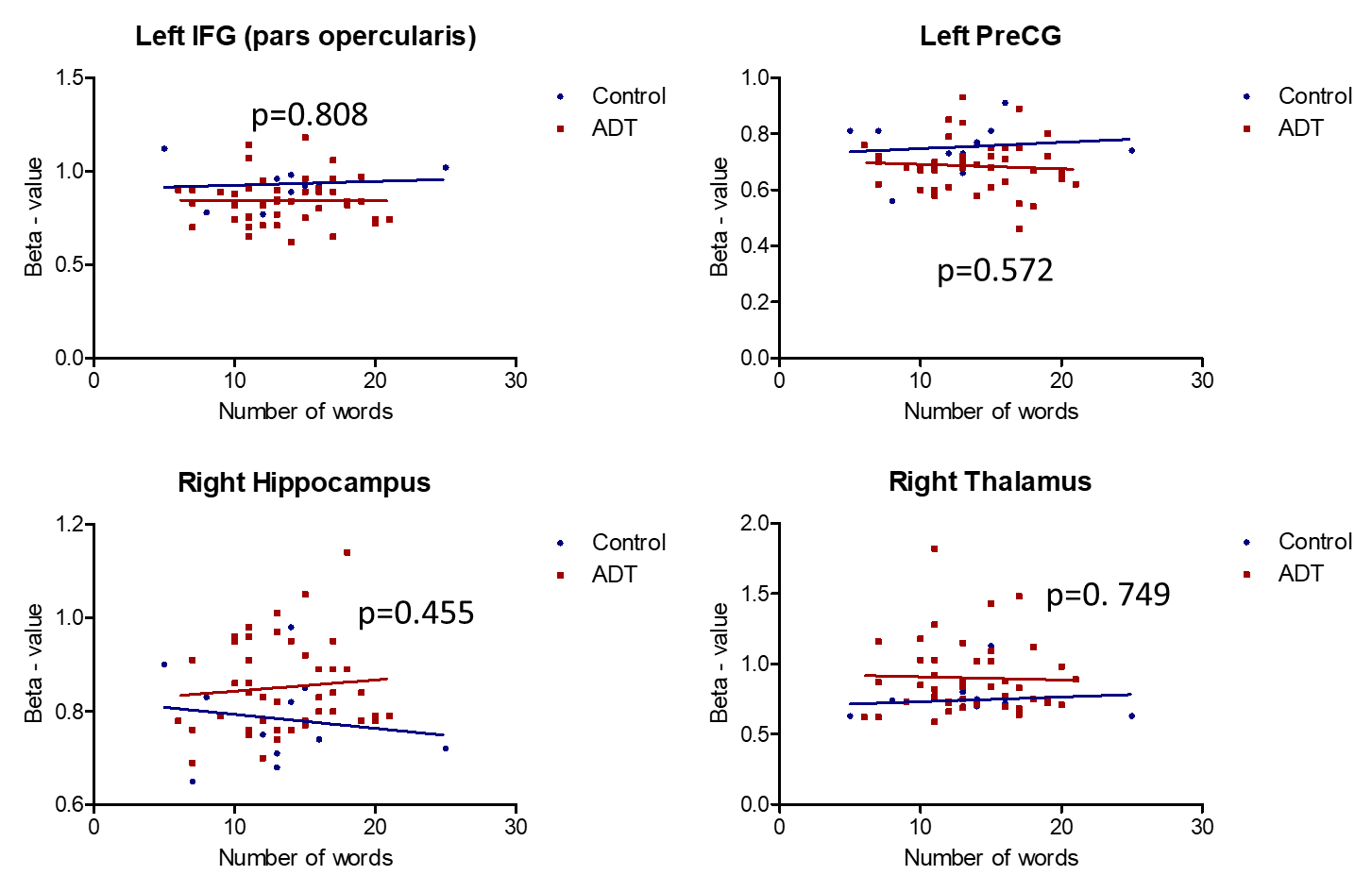


**Supplementary figure 3.** **Interaction analysis between ALFF in selected ROIs and Hooper Organization Visual Test (HOVT) score.**


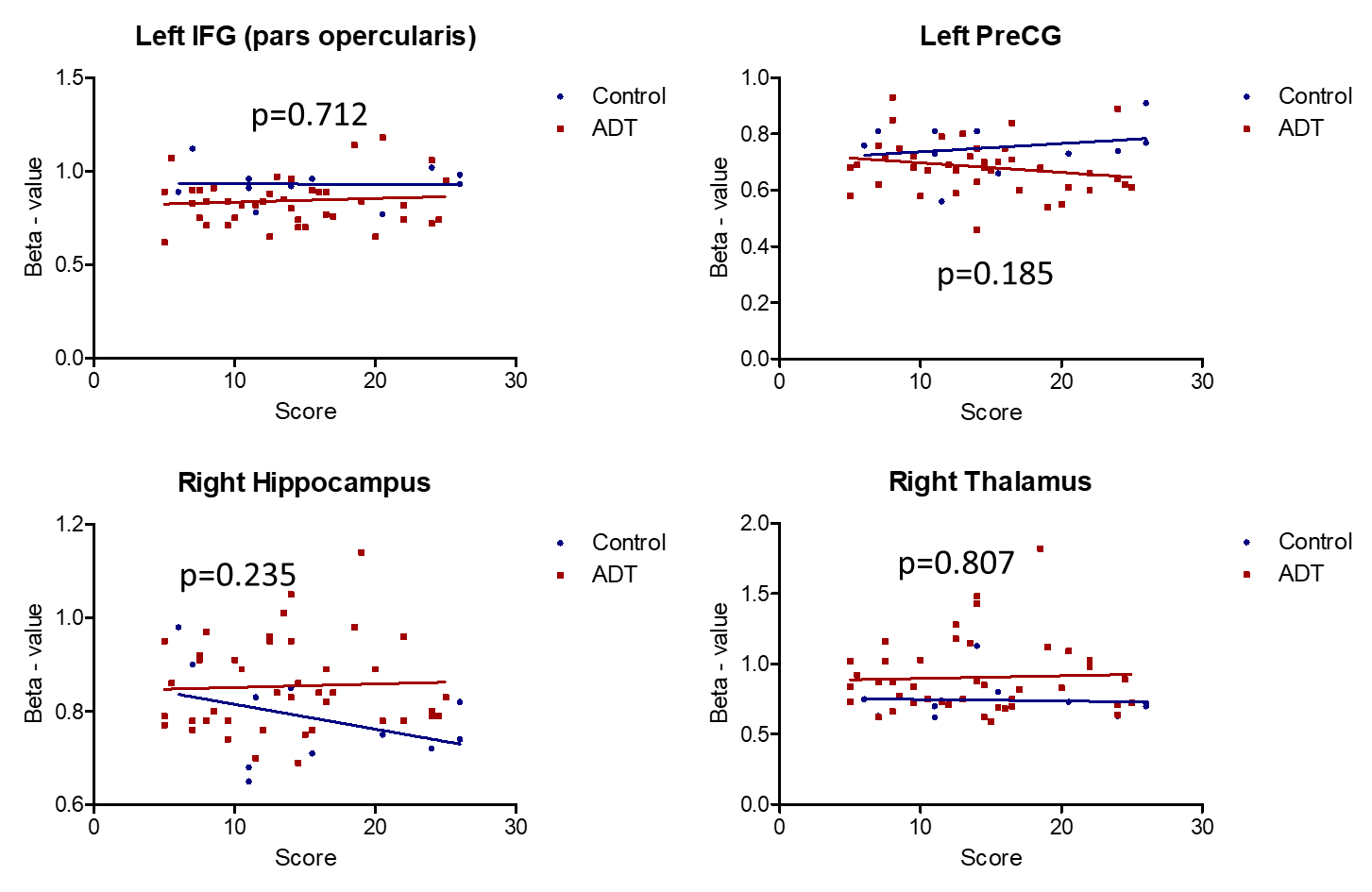


**Supplementary figure 4.** **Interaction analysis between ALFF in selected ROIs and Judgement Line Orientation Test (JLOT) score.**


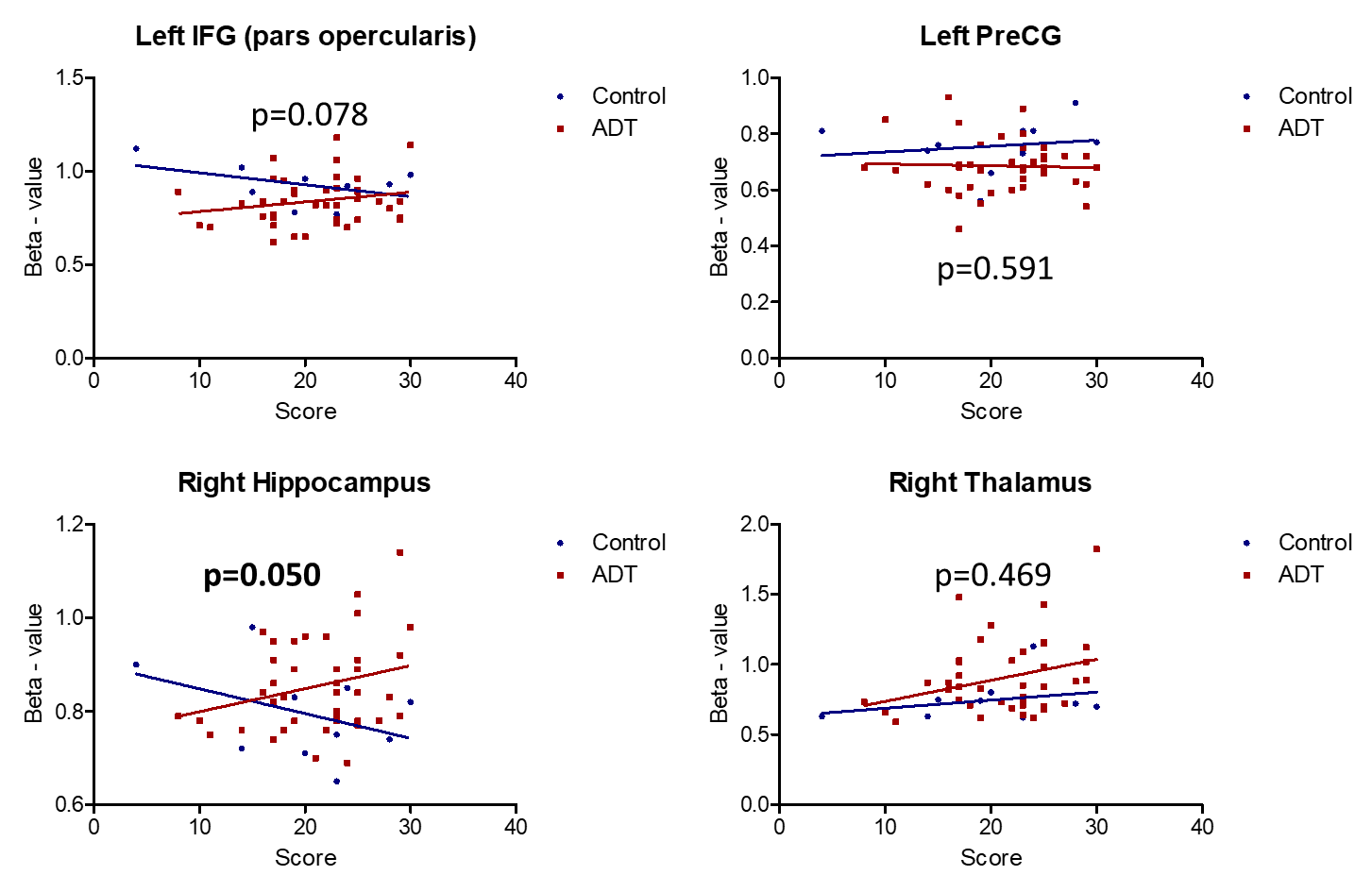


**Supplementary figure 5.** **Interaction analysis between ALFF in selected ROIs and Trail Making Test Part A (TMT A) score.**


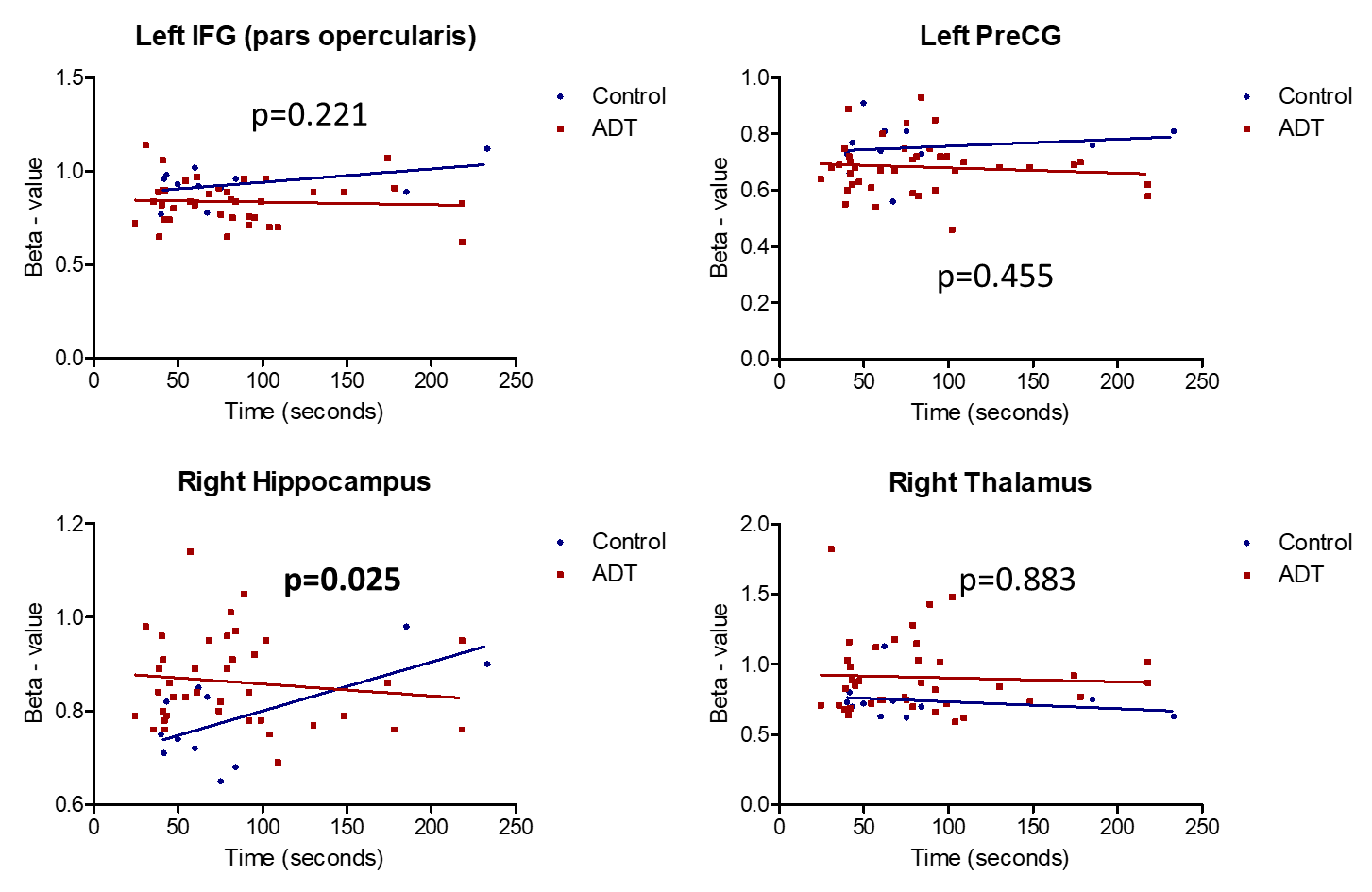


**Supplementary figure 6.** **Interaction analysis between ALFF in selected ROIs and Brief Visuospatial Memory Test (BVMT) score.**

**
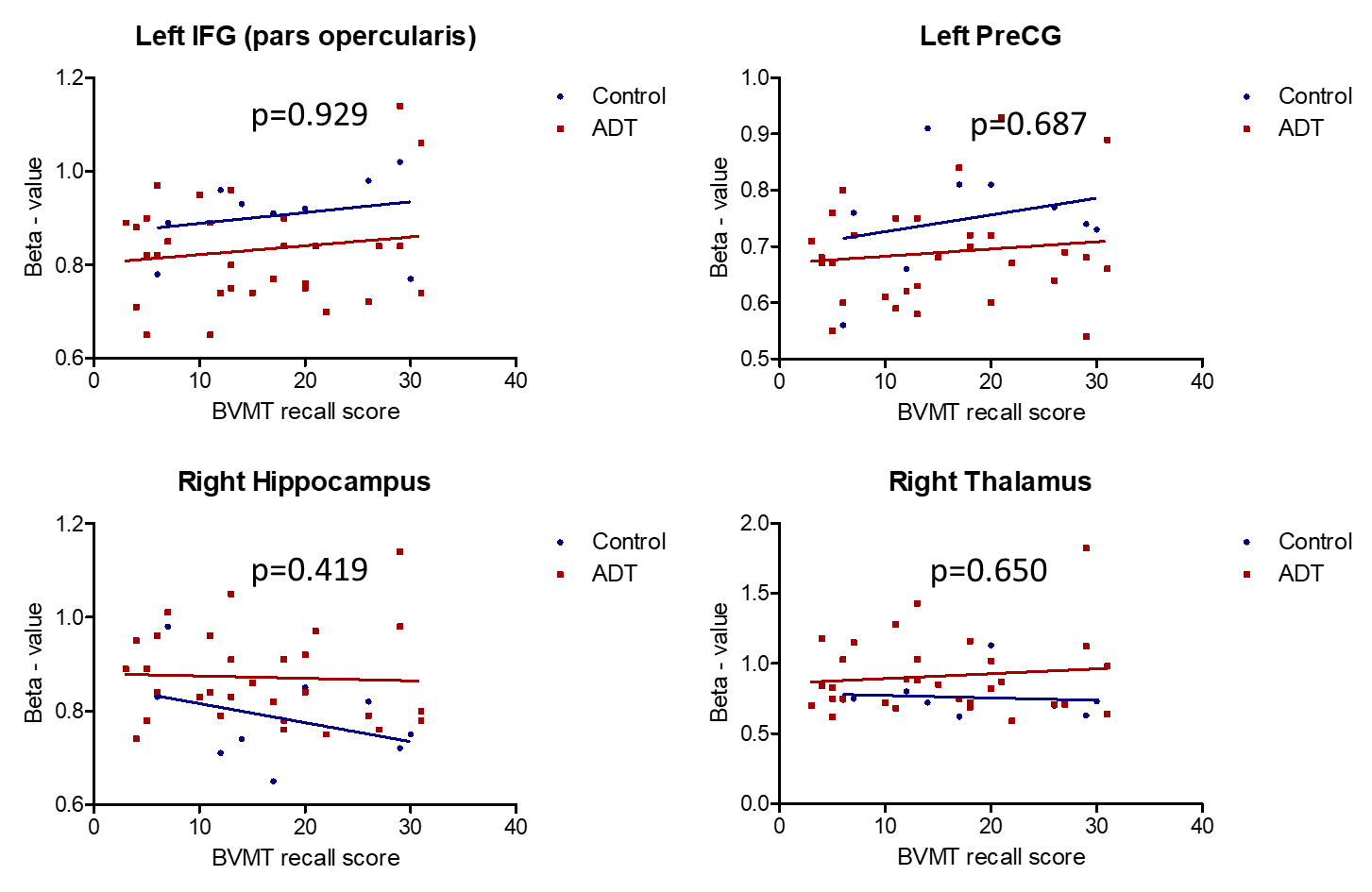
**

**Supplementary figure 7.** **Interaction analysis between ALFF in selected ROIs and Auditive Verbal Spanish Complutense Test (TAVEC) score.**


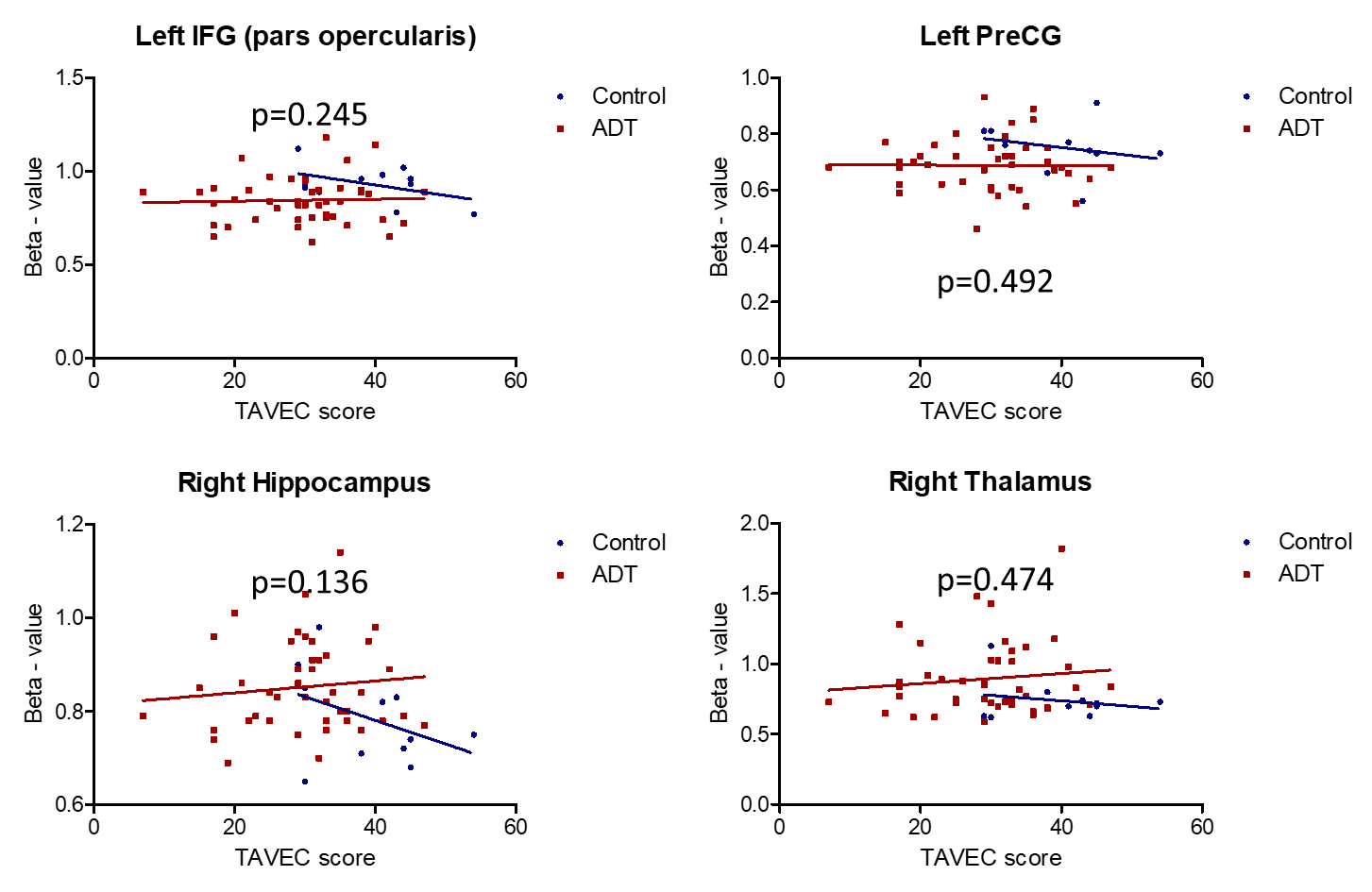

Supplement: Supplementary file 1 — Supplementary Information. [file 41598_2021_2611_MOESM1_ESM.docx]
